# Traumatic Adrenal Hemorrhage Masking as a Pseudotumor

**DOI:** 10.7759/cureus.7256

**Published:** 2020-03-13

**Authors:** Nandita Rao, Bracken Burns, Diane Cobble

**Affiliations:** 1 Surgery, East Tennessee State University, Johnson City, USA; 2 Surgery, Quillen College of Medicine, East Tennessee State University, Johnson City, USA; 3 Trauma Critical Care/Surgery, East Tennessee State University, Johnson City, USA

**Keywords:** traumatic adrenal hemorrhage, pseudotumor, adrenal, hypertension

## Abstract

Several case reports have been filed regarding the latent presentation of hemorrhagic pheochromocytomas in the trauma setting; however, few patients have been found to exhibit these symptoms in the absence of a tumor.

In this report, we discuss a patient who sustained blunt abdominal trauma leading to the development of an adrenal hemorrhage and his unexpected sequelae of symptoms. Discovery of the source of the patient's symptoms was delayed secondary to multiple comorbidities in the critical care setting and work-up for other sources such as infection and agitation. Hypertensive urgency was confirmed to be of adrenal etiology with measurement of persistently elevated plasma and urine metanephrines during the hospital course. The patients hypertensive urgency was successfully managed with the use of antisympathomimetics including an esmolol drip, clonidine, and eventually tapered dose of metoprolol. Symptoms improved over time, and repeat CT imaging weeks later showed resolution of the hematoma.

Review of literature reveals only one other case of adrenal hemorrhage after blunt force trauma resulting in hemorrhagic psuedotumor. To our knowledge, this is the second such case ever presented. This case is discussed along with the presentation, diagnostic work-up, and treatment of a critically ill patient with an adrenal hemorrhage masked as a pseudotumor.

## Introduction

Pheochromocytomas are rare endocrine tumors to encounter within the general patient population and even more uncommon in an acute trauma setting. The disease involves enterochromaffin cells within the adrenal gland medulla that lead to the overproduction of catecholamines and subsequent induction of the sympathetic nervous system. According to the literature, the incidental detection of pheochromocytomas has increased in otherwise asymptomatic patients based on the increased use of abdominal imaging during the management of trauma patients [1]. Although rare, it is more common to find incidental pheochromocytomas in the trauma setting than adrenal injuries that mimic pheochromcytomas. There is one known case of a patient who suffered a fall from a ladder and presented to a trauma center with arterial hypertension. An intra-adrenal hematoma was discovered, presenting itself as a hormonally active pheochromocytoma [2]. The rare case we will discuss presents not an incidental pheochromocytoma but rather a traumatic adrenal hemorrhage behaving as a pseudotumor. The patient exhibited typical symptomology of a pheochromocytoma and ultimately responded to treatment used to manage real tumor.

## Case presentation

A 33-year-old unrestrained male driver involved in a head-on, high-speed motor vehicle collision presented to Johnson City Medical Center in Johnson City, Tennessee as a level 1 trauma alert in February 2016. He was intubated in the field and per the emergency medical services report was initially hypertensive and tachycardic during transport. Vitals upon arrival on assist control ventilation were temperature (T) 95.3°F, heart rate (HR) 106 beats per minute, blood pressure (BP) 89/60 mmHg, and oxygen saturation (O2 sat) 100%. During primary survey, the patient was scored Glasgow Coma Scale 3T with multiple orthopedic injuries and a distended abdomen. Massive transfusion protocol was initiated. The patient became increasingly hypotensive with only transient improvement in mean arterial pressure. He was emergently transported to the operating room (OR) and underwent multiple small and large bowel resections, secondary to blunt force perforation and mesenteric bleeding. He was left in discontinuity and returned to the OR 24 hours postoperatively for creation of primary anastomoses and abdominal closure. At time of initial and subsequent operation, the adrenal hemorrhage was unknown, and with no evidence of expanding retroperitoneal hematoma this area was not explored. During the early resuscitation period, the patient exhibited both transient tachycardia and hypertension documented as HR 110 beats per minute and BP 165/67 mmHg on the morning of postoperative day 1. CT scan was obtained after initial stabilization and illustrated few small punctate cerebral hemorrhages, single non-displaced facial fracture, multiple extremity fractures, thoracic spinous process fractures, multiple rib fractures, and a hematoma of the right adrenal gland approximately 2.2 x 3.5 cm in size.

Despite overall improvement with resuscitation and routine postoperative care, the patient continued to exhibit persistent hypertension and tachycardia even with administration of continuous intravenous (IV) sedatives such as fentanyl and midazolam. Additional medications, including oxazepam and quetiapine, were added as clinical findings were thought to be concurrent with intermittent idiopathic episodes of agitation, but this only led to mild improvement. Pain control was also reassessed, and subjective patient reports indicated adequate management. Around hospital day 7, metoprolol was added to medication regimen, but despite increased dosage it only displayed a limited effect on hemodynamic state during the next 24 hours. The patient’s BP was continuously elevated with systolic blood pressure averaging 200 mmHg. IV sedation was switched to propofol in an attempt to alleviate persistent hypertensive urgency. At this time, work-up was also significant for the development of fever with worsening leukocytosis. The patient was 7-10 days postoperative and at high risk for possible intra-abdominal abscess or leak of intestinal anastomosis. Abdominal CT was obtained with overall unremarkable results showing stable right adrenal hematoma, development of small bilateral pleural effusions, and expected postoperative inflammatory changes.

Due to inadequate BP control, a continuous IV esmolol drip was started and both plasma and urine metanephrines were ordered. Labs illustrated plasma metanephrines at 0.26 and normetanephrine at 1.89 nmol/L (normal reference range 0-0.49 and 0-0.89 nmol/L, respectively). Urinary metanephrines were also elevated at 227 and normetanephrines at 1,159 μg/g (normal 0-300 and 0-400 μg/g, respectively) [1]. Esmolol drip was weaned within the next few days with addition of both clonidine and metoprolol. Vital signs at this time were documented around BP 170/80 mmHg and HR 120 beats per minute, and two days later recorded at BP 151/86 mmHg and HR 103 beats per minute. Recollection of labs, one week after initial values, showed persistently elevated urinary metanephrines and normetanephrines at 333 and 878 μg/g, respectively. BP was still elevated at this time but able to be controlled on oral agents alone; therefore, clonidine dose was tapered over the next few days. Maximum BP and HR values were recorded at 140/90 mmHg and 95 beats per minute, respectively, approximately one month into hospital stay. As the patient had otherwise recovered well from recent traumatic injuries, he was placed on a tapered dose of oral metoprolol (12.5 mg twice daily) and discharged home. The patient did not present for scheduled post hospital outpatient follow-up. The patient however did subsequently present to the hospital for unrelated reasons one month later (almost two months after initial admission date). At this presentation to the ED, there was no evidence of persistently elevated BP or HR, and repeat CT abdomen did not show any residual adrenal hemorrhage. Due to lack of follow-up, no further outpatient testing of metanephrines was able to be completed.

## Discussion

As per the literature, there is an increase identification of incidental pheochromocytomas during abdominal CT after traumatic injury [1]. A review of recent literature describes the most commonly accepted pathophysiology for traumatic adrenal stimulation to include a multifactorial causality. Physical injury may be a direct cause of transient catecholamines release into the bloodstream, but when combined with hypovolemic shock it can also create necrosis of the adrenal gland [3]. This tissue injury can produce a more significant release of plasma catecholamines to create a more profound or prolonged effect. Schmidt et al. concluded that increased intra-adrenal pressure induced reactive hyperplasia to the adrenal gland that lead to necrosis and release of excessive catecholamines in a patient who suffered adrenal injury from blunt force trauma to the abdomen from a fall [2]. Presentation is often latent due to time required to develop a sizeable hemorrhage, and severity of sequelae is also affected by the degree of overall injury [4].

This case illustrates the discovery of an adrenal injury as a direct cause of persistent tachycardia and hypertension in a trauma patient. Delay in identification of source was likely attributed to primary investigation of other more common etiologies, such as inadequate analgesia and an underlying infectious process. The expected progression of systemic inflammatory response syndromes and sepsis to affect the autonomic nervous system should always be carefully reviewed as either a sole cause or contributor to hemodynamic abnormality. Trauma patients may present with latent injuries or those not identified on initial physical exam due to distracting or more severe injures requiring immediate attention [5]. Based on this case presentation and the case published by Schmidt et al., providers should perhaps have a higher index of suspicion of possible adrenal etiology in trauma patients with adrenal hemorrhage and persistent tachycardia and hypertension.

Treatment options may vary based on extent of the injury with initial medical optimization and then conversion to operative management based on overall clinical progression. Based on the literature, the gold standard is α-adrenergic blockers or selective α-blockers as first-line drugs to control blood pressure and β-adrenergic blockers or selective β-blockers as second-line drugs to control heart rate [6]. It is usually recommended that hypertension in the acute trauma setting is treated medically as there is a higher risk of morbidity and mortality with early surgical intervention [7]. One specific study demonstrated that the mortality rate for patients who underwent emergent surgery prior to α-blockade had a mortality rate of 18%, whereas those who had elective/urgent surgery after initiation of α-blockade had a mortality rate of 0% [8]. If surgery is necessary, it appears that an elective surgery is optimal. In this case, surgery was not required and the patient's symptoms resolved after medical treatment. Insufficient data are available to specify a direct reason, but it may be presumed that avoidance of additional physical stress in a trauma patient with potentially multiple injured organ systems is optimal.

## Conclusions

This patient’s hospital course reflected adequate nonoperative management of his injury, and ultimately improvement of clinical symptomology with resolution of radiologic findings. Adrenal hemorrhage presenting as a pseudotumor is rare, with this being only second known case reported in the literature.
